# Live-Cell Assays for Cell Stress Responses Reveal New Patterns of Cell Signaling Caused by Mutations in Rhodopsin, α-Synuclein and TDP-43

**DOI:** 10.3389/fncel.2019.00535

**Published:** 2019-12-18

**Authors:** Kevin M. Harlen, Elizabeth C. Roush, Joseph E. Clayton, Scott Martinka, Thomas E. Hughes

**Affiliations:** ^1^Montana Molecular, Bozeman, MT, United States; ^2^BioTek Instruments Inc., Winooski, VT, United States

**Keywords:** neurodegeneration, Parkinson’s disease, unfolded protein response, retinitis pigmentosa, cAMP, Ca^2+^, biosensor, ER stress

## Abstract

Many neurodegenerative diseases induce high levels of sustained cellular stress and alter a number of cellular processes. To examine how different mutations associated with neurodegenerative disease affect cell stress and signaling, we created live-cell assays for endoplasmic reticulum (ER)-mediated cell stress and second messenger signaling. We first examined neurodegenerative mutations associated with direct ER stress by exploring the effect of rhodopsin mutations on ER stress and Ca^2+^ signaling. The rhodopsin P23H mutation, the most common mutation in autosomal dominant Retinitis Pigmentosa (RP), produced increased ER stress levels compared to wild type (WT) rhodopsin. Moreover, this increase in cell stress correlated with blunted Ca^2+^ signaling in a stress-dependent manner. Analysis of single-cell Ca^2+^ signaling profiles revealed unique Ca^2+^ signaling responses exist in cells expressing WT or P23H rhodopsin, consistent with the idea that second messenger signaling is affected by cell stress. To explore the use of the ER-stress biosensor in neurodegenerative diseases that may not have a direct effect on ER-mediated cell stress, we examined how various mutants of α-synuclein and TDP-43 affected ER stress. Mutants of both α-synuclein and TDP-43 associated with Parkinson’s disease (PD) and Amyotrophic lateral sclerosis (ALS) demonstrated increased ER stress compared to WT proteins. To examine the effect of α-synuclein and TDP-43 mutants on cellular signaling, we created a second live-cell assay to monitor changes in cAMP signaling during expression of various forms of α-synuclein and TDP-43. The increased cell stress caused by expression of the mutant proteins was accompanied by changes in phosphodiesterase activity. Both HEK293T and SH-SY5Y cells expressing these proteins displayed a shift towards increased cAMP degradation rates, likely due to increased phosphodiesterase activity. Together these data illustrate how biosensors for cellular stress and signaling can provide nuanced, new views of neurodegenerative disease processes.

## Introduction

Genetically-encoded fluorescent biosensors are powerful tools that have provided new views of how circuits in the brain operate, and how cells and networks of cells process and respond to stimuli (Chen et al., 2017). Many of these biosensors detect small molecule analytes, like Ca^2+^, cyclic AMP (cAMP) and diacylglycerol (Zhao et al., 2011; Tewson et al., 2012, 2016; Broussard et al., 2014) that change in concentration during cell signaling events. Biosensors of this type are used to monitor events such as G-protein coupled receptor (GPCR) activation or neuronal Ca^2+^ signaling. Because they are protein-based, biosensors can be targeted to distinct sub-populations of cells or to specific organelles and subcellular domains (Moore et al., 2016; Pendin et al., 2017), and can be systemically delivered by viral vectors. These features have popularized genetically-encoded biosensors, especially those for Ca^2+^ and cAMP (Castro et al., 2014), for use in both *in vitro* as well as *in vivo* models (Chen et al., 2017). Another type of genetically encoded biosensor targets changes to the state of the cell. For example, biosensors for apoptosis (Xu et al., 1998), cell cycle state (Sakaue-Sawano et al., 2008), autophagy (Katayama et al., 2011), and cell stress (Iwawaki et al., 2004; Roy et al., 2017) have been developed to detect broad changes to cellular states. However, these two classes of biosensors are often used in separate assays to examine unique outcomes of either change in cell state or signaling. We reasoned that combining biosensors for cell state with those for cell signaling could provide new insights as to how changes in cell state, such as cell stress, alter cellular signaling.

Neurodegenerative disorders, such as Parkinson’s disease (PD), Amyotrophic lateral sclerosis (ALS), and the degenerative blinding disease Retinitis Pigmentosa (RP) all involve cellular stress and occur over the course of many years. Each disease is also linked to changes in second messenger signaling. In RP, rod photoreceptors slowly degrade over time, eventually degrading the cone photoreceptors as well, leading to photoreceptor cell death and blindness (Hartong et al., 2006; Ferrari et al., 2011; Koch et al., 2015). The most common mutation associated with RP is the autosomal dominant P23H mutation within the rhodopsin gene (Ferrari et al., 2011). This rhodopsin mutation is accompanied by changes in Ca^2+^ and cyclic GMP (cGMP) signaling, along with increased cell stress triggered by the unfolded protein response (UPR; Arango-Gonzalez et al., 2014; Shinde et al., 2016). In Parkinson’s and ALS, unique subpopulations of neurons experience prolonged cell stress before eventually dying (Bosco et al., 2011; Taylor et al., 2016; Maiti et al., 2017). Parkinson’s and ALS are characterized by the accumulation of misfolded proteins throughout the cell. However, there is also evidence that the modulation of second messenger signaling levels mediated through GPCR activity may influence disease progression (Xu et al., 2012; Mittal et al., 2017). Furthermore, inhibition of phosphodiesterase activity, which is responsible for the breakdown of cAMP and cGMP, has been demonstrated to preserve dopaminergic neurons in models of PD (Morales-Garcia et al., 2011). Thus, accumulating evidence suggests that changes in both cell stress and signaling are associated with multiple neurodegenerative diseases.

Imagine the neuron suffering under the load of a misfolded protein for years on end. Which stress pathways are activated and how does it compensate for the stress? Does it still respond to its environment appropriately? Can it still sense the same neurotransmitters and neuromodulators in the same way? Does it still respond to drugs in the same way that the healthy, surrounding cells do? To answer these questions live-cell assays conducted in models of neurodegeneration must be developed. Genetically-encoded biosensors represent useful tools for the development of these assays because of their ability to monitor cellular function in real-time. Multiple biosensors can also be paired to monitor different cellular activities and pathways simultaneously, providing details of cellular function that cannot be assessed by simply monitoring cell death.

Endoplasmic reticulum (ER) stress is associated with multiple neurodegenerative diseases including RP, Parkinson’s, and ALS (Shinde et al., 2016; Remondelli and Renna, 2017) and occurs well before cell death, making it an important live-cell assay target. We created an ER-stress biosensor that can be combined with biosensors for second messengers. We then monitored, either simultaneously or orthogonally, changes in cell stress levels and the effects on cellular signaling. To analyze these signaling changes we created two assays. We first examined how the expression of mutant rhodopsin affects ER-stress and Ca^2+^ signaling. We show that a blunted Ca^2+^ signaling response accompanies increased ER stress in cells expressing the rhodopsin P23H mutant. Next, using mutants of α-synuclein and TAR DNA binding protein (TDP-43) we created a second live-cell assay that reveals how cAMP signaling is altered under ER stress brought on by expression of these neurodegenerative associated proteins. Together, these results demonstrate the usefulness of live-cell assays utilizing unique genetically-encoded fluorescent biosensors as models to study neurodegenerative disease.

## Materials and Methods

### Biosensor Construction

**Figure 1:**
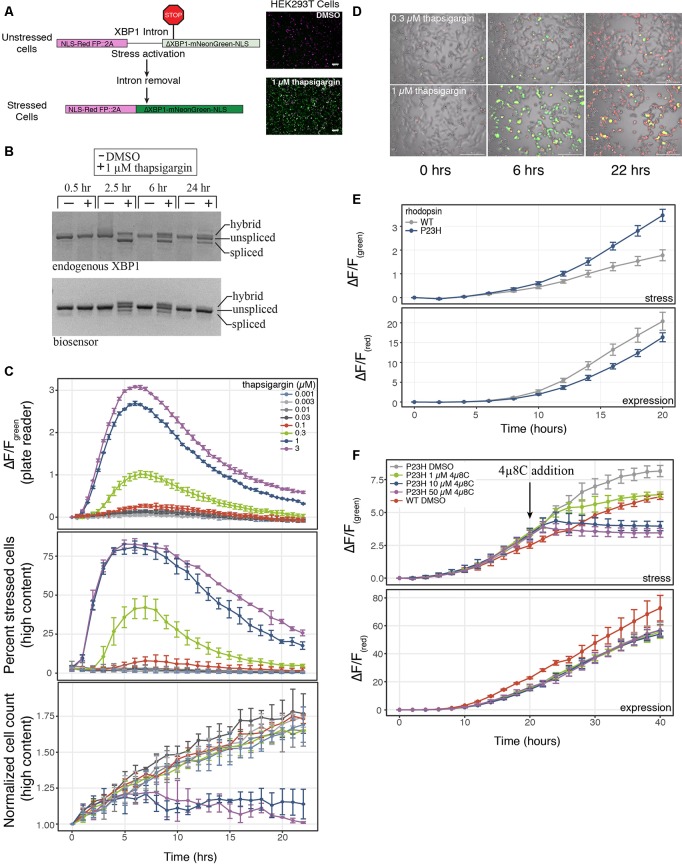
A genetically-encoded fluorescent biosensor to detect endoplasmic reticulum (ER)-mediated cell stress. **(A)** Schematic of the two-color cell stress biosensor. The biosensor is polycistronic transcript consisting of a nuclear targeted constitutively expressed red fluorescent protein followed by a self-cleaving 2A peptide and a stress-induced nuclear targeted green fluorescent protein (mNeonGreen) fused to a portion of the XBP1 protein (ΔXBP1-mNeonGreen). Activation of ER stress through the IRE1α-XBP1 pathway splices out a portion of the XBP1 mRNA, shifting mNeonGreen into frame. Top: unstressed cells; bottom: stressed cells treated with 1 μM thapsigargin. Scale bar = 100 μm. **(B)** Reverse-transcription polymerase chain reaction (RT-PCR) agarose gel of the splicing status of either endogenous or biosensor XBP1 transcripts over 24 h. Cells were treated with either DMSO or 1 μM thapsigargin. Three bands are present, unspliced XBP1, spliced XBP1 and a hybrid band of spliced and unspliced transcripts (Chalmers et al., 2017). **(C)** HEK293T cells were transduced with the cell stress biosensor and treated with increasing amounts of thapsigargin. Top: the fold change (ΔF/F) in green fluorescence was calculated over 22 h, measured every 30 min on a plate reader. Middle: the percent of stressed cells per image was calculated every hour on a high content imager. Bottom: the normalized cell count per image was calculated every hour on a high content imager. Data are plotted as mean × SD, *N* = 3 wells per condition. **(D)** Overlay of phase, red fluorescence, and green fluorescence images of HEK293 cells transduced with the cell stress biosensor and treated with 0.3 μM and 1 μM thapsigargin. Images acquired at 0, 6 and 22 h after treatment, scale bar = 200 μm. **(E)** HEK293T cells were transfected with the cell stress biosensor and either wild type (WT) or mutant P23H rhodopsin. The fold change in green and red fluorescence ratio is plotted over 20 h. Fold change in stress response (ΔF/F_green_) and expression (ΔF/F_red_) is shown. Data are plotted as mean × SD, *N = 3* wells per condition. **(F)** After 20 h the cells from **(E)** were treated with increasing amounts of the IRE1α inhibitor 4μ8C or DMSO. The fold change in stress response (ΔF/F_green_) and expression (ΔF/F_red_) were monitored for an additional 20 h.

The ER-stress biosensor was modified from Iwawaki et al. (2004). mNeonGreen was placed downstream of the XBP1 sequence and splice site. SantakaRFP (ATUM FPB-58-609) was placed upstream of the XBP1 sequence (Figure 1A). The SantakaRFP and XBP1-mNeonGreen sequences were separated by a self-cleaving 2A peptide sequence. For the R-GECO-cell stress biosensor SantakaRFP was replaced with R-GECO1.2 (Wu et al., 2013; Figure 3A). Cloning was done using In-Fusion HD cloning (Takara 638911). R-GECO (U0600R), R-cADDis (U0200R) and bPAC (V0100N) were obtained from Montana Molecular.

**Figure 2:**
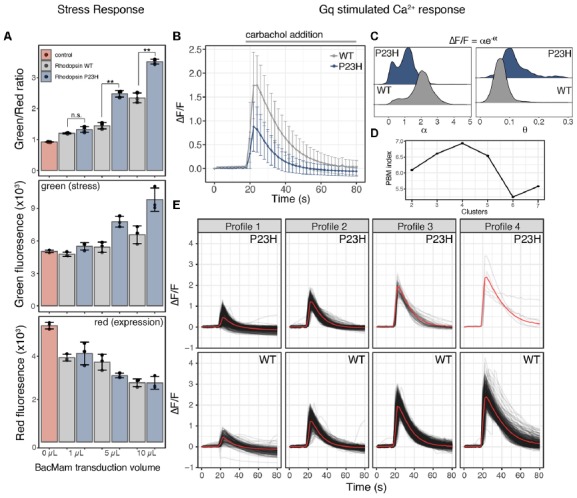
Mutant P23H rhodopsin elicits ER-mediated cell stress and alters Ca^2+^ signaling. **(A)** HEK293T cells transduced with the cell stress biosensor and either no protein (control), wild type (WT) rhodopsin or P23H rhodopsin. After 24 h the green, red, and green/red fluorescence ratio were analyzed. Data are plotted as mean ± SD, *N* = 3 wells per condition, **P-value < 0.01, n.s. = not significant. **(B)** Average profiles of the fold change in cytoplasmic Ca^2+^ in cells expressing the R-GECO Ca^2+^ biosensor and transduced with 5 μl WT or P23H rhodopsin during stimulation with 30 μM carbachol. Data are plotted as the mean ± SD of two wells and 2,125 cells for WT and two wells and 1,023 cells for P23H. **(C)** Ca^2+^ signaling profiles form individual cells in **(B)** expressing WT or P23H rhodopsin were fit to equation ΔF/F = αe^−θt^, where ΔF/F is the fold change in fluorescence, α is the signaling amplitude, θ is the exponential decay rate, and t is time in seconds. Density plots of the values for α and θ from each cell are shown for WT and P23H expressing cells. **(D)** PBM index score of Ca^2+^ signaling profile clusters in WT and P23H rhodopsin expressing cells. A maximum at four clusters indicates Ca^2+^ signaling profiles can be best segmented into four distinct profiles. **(E)** Individual Ca^2+^ responses from each cell in **(B)** were analyzed and separated into four distinct profiles based on the PBM index score from **(D)**. Each black line represents a single cell; the red line represents the mean response of each profile.

**Figure 3:**
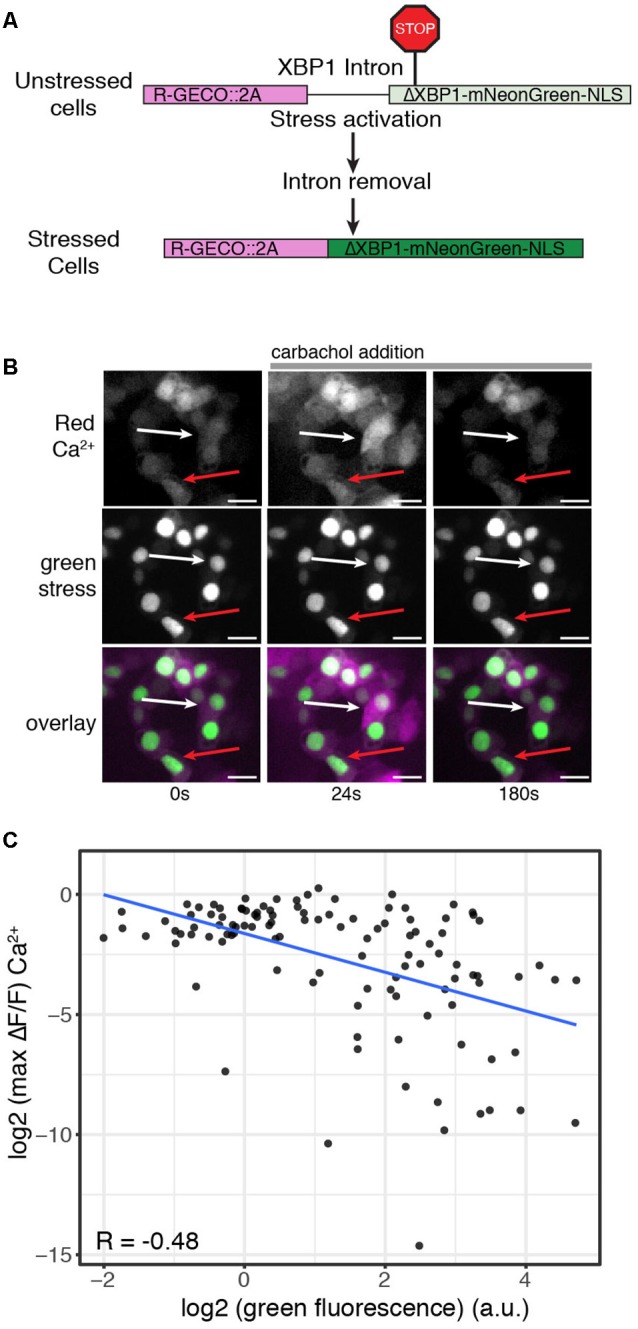
ER stress levels correlate with decreased Ca^2+^ signaling response. **(A)** Schematic of the R-GECO cell stress biosensor. **(B)** Representative image of cells expressing the R-GECO cell stress biosensor and the rhodopsin P23H mutant. Top: red Ca^2+^ channel, middle: green stress channel, bottom: merge. Images are taken before during and after carbachol stimulation. The white arrow depicts a lowly stressed cell. The red arrow depicts a highly stressed cell. Scale bar = 25 μm. **(C)** Scatter plot comparing the stress levels and max fold change in Ca^2+^ levels of individual cells expressing the R-GECO cell stress biosensor and rhodopsin P23H. The blue line is the linear correlation between the two variables. R = Pearson correlation coefficient.

### Chemicals

Sodium butyrate (B5887), valproic acid (P4543), and carbachol (C4382) were obtained from Millipore Sigma. Thapsigargin (10522) and 4μ8C (22110) were obtained from Cayman Chemical.

### Cell Culture

**Figure 4:**
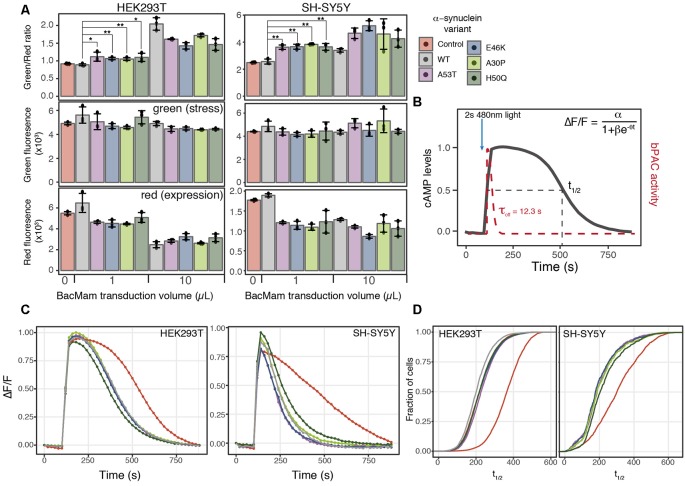
α-synuclein expression induces ER-mediated cell stress and alters PDE activity. **(A)** HEK293T or SH-SY5Y cells were transduced with the cell stress biosensor and either no other virus (control) or 1 or 10 μl of WT α-synuclein or one of four α-synuclein mutants. The green, red, and green/red ratio fluorescence were measured after 24 h of expression. Individual data points are plotted and bars represent the mean, error bars represent the standard deviation of *N* = 3 wells. **(B)** Schematic of optical PDE activity assay. Cells were transduced with the red fluorescent cAMP biosensor R-cADDis and the blue light-activated adenylyl cyclase bPAC. Baseline cAMP levels were monitored prior to bPAC activation to raise cAMP levels. A 2-s pulse of blue light activates bPAC to raise cAMP. bPAC activity decays rapidly and cAMP levels were monitored for 15 min at which point cAMP levels return to baseline. cAMP degradation profiles were fit with the following logistic function ΔF/F = α/1+βe^−θt^ for cAMP half-life calculation where ΔF/F is the change in fluorescence, α is the maximum cAMP level, βe^−\rtheta^ is the exponential decay rate, and t is time in seconds. **(C)** Average cAMP degradation profiles in HEK293T and SH-SY5Y cells expressing different variants of α-synuclein or control cells not over-expressing any form α-synuclein. For HEK293T cells *N* = 3 wells per sample, control = 4,047 cells, WT = 3,882 cells, A53T = 3,385 cells, E46K = 3,431 cells, A30P = 3,700 cells, H50Q = 3,455 cells. For SH-SY5Y cells *N* = 3 wells per sample, control = 2,697 cells, WT = 2,427 cells, A53T = 2,775, E46K = 1,462 cells, A30P = 1,902 cells, H50Q = 2,209 cells. **(D)** Cumulative distribution functions plotted for the distribution of cAMP half-lives (t_1/2_) determined from the individual cells analyzed in **(C)**. *P-value < 0.05, **P-value < 0.01.

**Figure 5:**
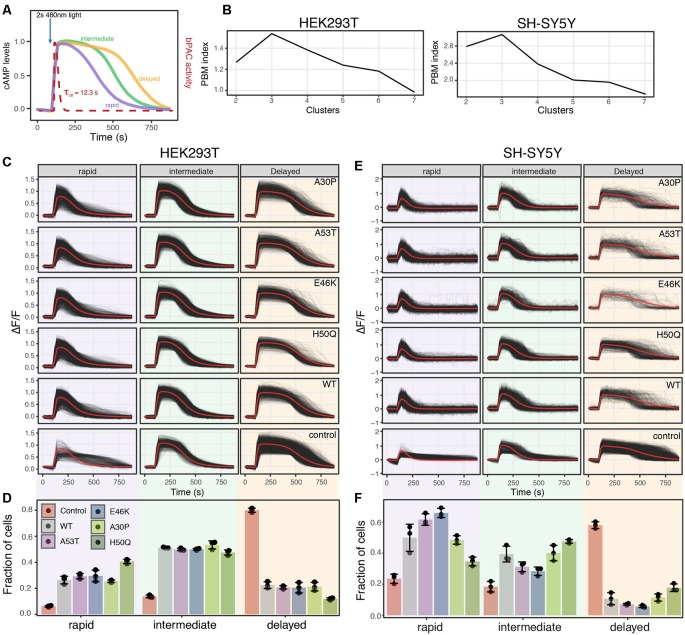
Analysis of PDE activity from individual cells reveals distinct PDE activity profiles. **(A)** Schematic of PDE activity profiles from individual cells revealed three distinct cAMP decay profiles, rapid, intermediate, and delayed. **(B)** PBM index score of cAMP degradation profile clusters in HEK293T and SH-SY5Y cells. Data from control cells or cells expressing WT or a mutant variant of α-synuclein were merged for analysis. A maximum at three clusters indicates cAMP degradation profiles can be best segmented into three distinct profiles for both HEK293T and SH-SY5Y cells. **(C)** Individual cAMP degradation profiles from control HEK293T cells and HEK293T cells expressing WT or a mutant variant of α-synuclein. Each black line represents a single cell; the red line represents the mean response of each profile. *N* = 3 wells per sample, control = 4,047 cells, WT = 3,882 cells, A53T = 3,385 cells, E46K = 3,431 cells, A30P = 3,700 cells, H50Q = 3,455 cells. **(D)** Bar plot of the fraction of cells in each profile for control and α-synuclein variants from the profiles determined in **(C)**. *N* = 3 wells for each condition. Individual data points are plotted along with the mean bar and error bars representing the standard deviation. Panels **(E,F)** same as in **(C,D)** but for SH-SY5Y cells. *N* = 3 wells per sample, control = 2,697 cells, WT = 2,427 cells, A53T = 2,775, E46K = 1,462 cells, A30P = 1,902 cells, H50Q = 2,209 cells.

**Figure 6:**
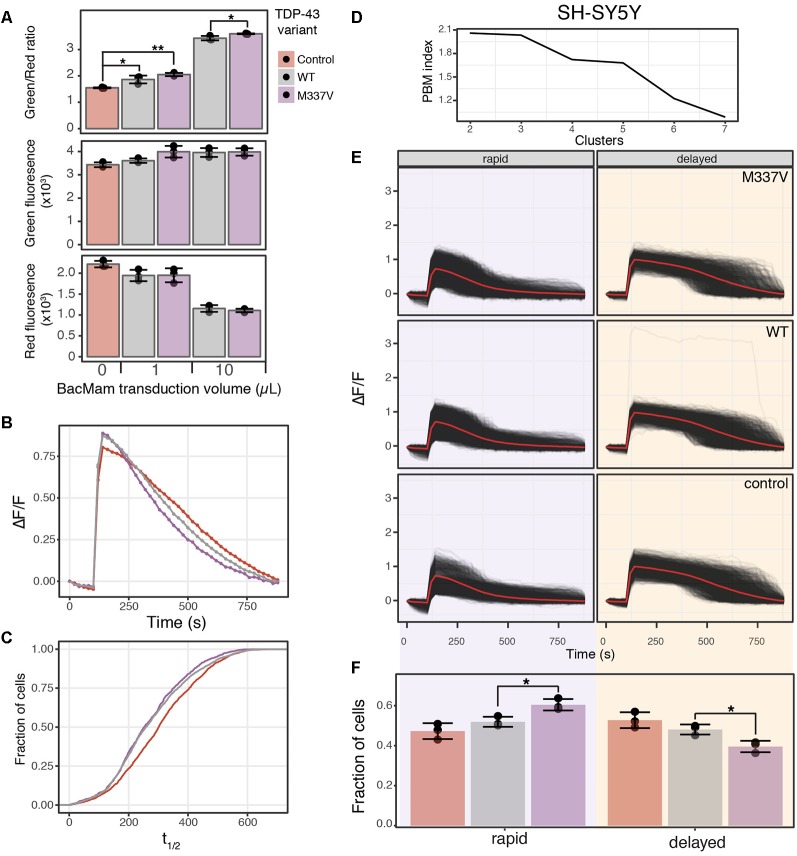
TDP-43 expression induces cell stress and increases cAMP degradation rate. **(A)** SH-SY5Y cells were transduced with the cell stress biosensor and either no other virus (control), 1 or 10 μl of WT or M337V TDP-43 virus. The green, red and green/red ratio fluorescence was measured after 24 h of expression. Individual data points are plotted and bars represent the mean, error bars represent the standard deviation of *N* = 3 wells. **(B)** Average cAMP degradation profiles in SH-SY5Y cells expressing different variants of TDP-43 or control cells. *N* = 3 wells per sample, control = 2,697 cells, WT = 3,352 cells, M337V = 3,023 cells. **(C)** Cumulative distribution functions plotted for the distribution of cAMP half-lives (t_1/2_) determined from the individual cells analyzed in **(B)**. To determine T_1/2_ cAMP, data from each cell were fit to the following equation ΔF/F = α/1+βe^−θt^ with the same parameters as Figure 4B. **(D)** PBM index score of cAMP degradation profile clusters in SH-SY5Y cells. Data from control cells or cells expressing WT or M337V versions of TDP-43 were merged for analysis. A maximum at two clusters indicates cAMP degradation profiles can be best segmented into two distinct profiles. **(E)** Individual cAMP degradation profiles from control cells and cells expressing WT or M337V TDP-43. Each black line represents a single cell; the red line represents the mean response of each profile. Well and cell number are the same as **(B)**. **(F)** Bar plot of the fraction of cells in each profile for control and TDP-43 variants from the profiles determined in **(E)**. *N* = 3 wells for each condition. Individual data points are plotted along with the mean bar and error bars representing the standard deviation. *P-value < 0.05, **P-value < 0.01.

HEK293T cells were obtained from ATCC (CRL-11268) and cultured in T-75 flasks at 37°C and 5% CO_2_ in EMEM media (ATCC 30-2003) containing 1× Penicillin-Streptomycin (Thermo Fisher Scientific 15140122), supplemented with 10% fetal bovine serum (FBS). SH-SY5Y cells were obtained from ATCC (CRL-2266) and cultured the same as the HEK293T cells except DMEM (ATCC 30-2002) which was used as the base media. For plate reader, experiments cells were cultured in Fluorobrite DMEM (Thermo Fisher Scientific A1896701) containing 4 mM Glutamax (35050061) and 10% FBS (Figure 1), or the media was replaced with DPBS (Figures 2–6). For imaging media was replaced with DPBS containing calcium and magnesium (Thermo Fisher Scientific SH30264.01). For all experiments cells were subcultured at 70–90% confluence and plated onto 96-well poly-D lysine coated microplates (Greiner Bio-One 82050-806).

### Gene Expression

Plasmid transfections were done using Lipofectamine 2000 (Thermo Fisher Scientific 11668019) according to the manufacturer’s instructions. Briefly, 0.4 μl of Lipofectamine 2000 per 100 ng of plasmid DNA was added to Opti-Mem reduced serum media (Thermo Fisher Scientific 31985088) to create a final volume of 100 μl. After incubation at room temperature for 20 min all 100 μl of the transfection mix was mixed with 100 μl of HEK293T cells and plated onto a single well of a 96-well microtiter plate. For all transfection experiments, 100 ng of either the cell stress biosensor or the R-GECO-cell stress biosensor plasmids were delivered along with 100 ng of WT or mutant P23H rhodopsin plasmid per 27,000 cells. Four hours later the media was exchanged for 150 μl of Fluorobrite DMEM media. For viral transduction experiments, the following BacMam viruses were used: cell stress biosensor (2 × 10^10^ viral genes (VG)/ml), R-GECO (3.1 × 10^10^ VG/ml), bPAC (1.18 × 10^11^ VG/ml), R-cADDis (5.06 × 10^10^ VG/ml), rhodopsin, a-synuclein, and TDP-43 viral titers listed in Table 1. For experiments comparing WT and mutant versions of rhodopsin, α-synuclein, and TDP-43 the viral volume was matched to the titer of the WT protein in the case of rhodopsin and TDP-43 and the A53T mutant of α-synuclein. For all experiments using BacMam 25 μl of biosensor was transduced per 48,000 cells. When bPAC was used it was transduced at 5 μl per 48,000 cells. For HEK293T cells all transductions were conducted in the presence of 2 mM sodium butyrate and for SH-SY5Y cells all transductions were conducted in the presence of 6 mM valproic acid. After 24 h incubation, the cells were analyzed for cell stress, Ca^2+^ signaling, or cAMP degradation.

**Table 1:** mRNA transcripts per cell for WT and mutant versions of the genes used in this study.

Protein	Mutation	Viral Titer (VG/ml)	Transduced virus (μl)	mRNA transcripts per cell	**TPM HEK293	Transcripts per cell HEK293	**TPM SH-SY5Y	Transcripts per cell SH-SY5Y
Rhodopsin	WT	6.73 × 10^10^	1	118.82	*5,592.8	1,118.56	0	0
			10	1,547.71
	P23H	6.50 × 10^10^	1	65.34
			10	2,103.75
α-synuclein	WT	5.00 × 10^10^	1	29.17	21.9	4.38	16.2	3.24
			10	667.60
	A53T	7.66 × 10^10^	1	139.01
			10	1,067.81
	A30P	7.93 × 10^10^	1	82.52
			10	989.90
	E46K	8.57 × 10^10^	1	62.86
			10	1,147.50
	H50Q	9.18 × 10^10^	1	101.65
			10	917.29
TDP-43	WT	8.77 × 10^10^	1	45.51	151	30.2	167.7	33.54
			10	58.62
	M337V	7.66 × 10^10^	1	36.11
			10	137.77				

All genes were packaged in BacMam and the viral titer was determined as viral genes (VG) per milliliter. mRNA transcripts per cell were determined by qRT-PCR and compared to endogenous expression levels in HEK293T and SH-SY5Y cells. *For rhodopsin expression data from the retina was used. **Data obtained from Uhlén et al. (2015). TPM, transcripts per million.

### RT-qPCR

Two wells of a 96-well plate containing 48,000 HEK293T (96,000 total) cells were transduced with either 1 or 10 μl of BacMam virus indicated in Table 1. Cells were then incubated for 24 h followed by RNA isolation using *Quick*-RNA Microprep Kit (Zymo Research R1050). cDNA was generated using the M-MLV Reverse Transcriptase kit (Promega M1701) according to the manufacturer’s instructions. Quantitative polymerase chain reaction (qPCR) was conducted using Syber select master mix for CFX (Thermo Fisher Scientific 4472942).

### Plate Reader, Imaging and Image Analysis

For the long-term plate reader analyses in Figure 1, cells were transfected as described above with the addition of 25 mM HEPES (Thermo Fisher Scientific 15630080) to the media. Cells were analyzed for 20–40 h on a BMG CLARIOstar (Figure 1C) or BioTek SynergyMX (Figures 1E,F) plate reader, heated to 37°C. Reads with excitation/emission of 485/528 and 558/603 with 20 nm bandpass were taken every 30 min. Data are plotted every 2 h for clarity. High content analysis (Figures 1C,D) was conducted on the Lionheart FX Automated Microscope (BioTek Instruments) at 37°C and 5% CO_2_. HEK293 cells were transduced with 10 μl of the cell stress biosensor per well in Fluorobrite DMEM containing 6 mM valproic acid and plated onto 96-well plates. Twenty-four hours after plating, increasing concentrations of thapsigargin was added to the cells and cells were imaged every hour using a 10× objective and the following filter sets, red fluorescence: 531 Ex, 593 Em LED filter cube, green fluorescence: 469 Ex, 525 Em LED filter cube. Images for Figure 2 were collected on a Zeiss Axiovert 200 using an Olympus UPlanFL 10×/0.30 lens and a Teledyne Qimaging 2000R CCD camera. Images were taken every 2 s exciting for 200 ms with a 1A 560 nm LED (ThorLabs DC4100) with a 556/20 Semrock excitation filter and a 617/70 Semrock emission filter. Images for Figure 3 were acquired on an Olympus IX81 using an Olympus UPlanSApo 20×/0.75 lens and a Hamamatsu C9100 EM-CCD camera. Images were acquired every 2 s exciting for 200 ms with a 1A Halogen Exfo X-cite series 120 lamp using the same excitation and emission filters. For all bPAC-RcADDis imaging in Figures 4–6, the Zeiss Axiovert with the 10× lens was used. Images were acquired every 20 s using a 100 ms exposure and the 556/20 Semrock excitation filter. After 100 s, the sample was pulsed with blue light for 2 s using a 1A 470 nm LED and a 470/20 Semrock excitation filter. Following this, pulse images were acquired every 20 s as before the pulse. All image analysis was conducted using CellProfiler (McQuin et al., 2018). Raw images and CellProfiler scripts are available in the supplemental materials.

### Statistical Analysis

Where applicable, data are reported as mean ± SD of at least three replicates. Either the fold change (ΔF/F) in fluorescence or the percent stressed cells was used to determine cell stress response. ΔF/F refers to the fold change in green fluorescence induced by activation of the stress sensor. Percent stressed cells is calculated by dividing the number of cells that display both green and red fluorescence by the total number of cells expressing red fluorescence within a single image. The percent stressed cell analysis was used to analyze high content data in Figure 1. For image analysis in Figures 2–6, individual cells from two or three images were analyzed, with the total number of wells and cells listed for each experiment in the figure legends. *P*-values were determined using two-tailed equal variance *t*-tests. Ca^2+^ signaling and cAMP degradation profile analysis was conducted in R^1^ using the TSrepr package (Laurinec, 2018). Data were first filtered for a positive increase in cAMP levels after bPAC stimulation by removing cells displaying a ΔF/F of less than 0.2. The number of cAMP degradation profiles was determined using the PBM index (Pakhira et al., 2004), identifying the number of clusters that had highest PBM index score. Calculation of the Ca^2+^ signaling amplitude and decay were conducted in R. Data from each cell was fit to the following exponential equation, ΔF/F = αe^−θt^, where ΔF/F is the fold change in fluorescence, α is the signaling amplitude, θ is the exponential decay rate, and t is time in seconds. The distribution of values for α and θ in cells expressing WT and P23H rhodopsin were then plotted. cAMP average profiles for each sample were calculated by average individual cAMP signaling profiles from each cell from three biological replicate images. cAMP half-lives were calculated by first fitting each cAMP degradation profile to following logistic function, ΔF/F = α/(1+βe^−θt^), where ΔF/F is the change in fluorescence, α is the maximum cAMP level, βe^−\rtheta^ is the exponential decay rate, and t is time in seconds. The t_1/2_ was then calculated for each cell from three biological replicate images. The cumulative distribution function of t_1/2_ values from each sample was then calculated in R and compared between samples. Note, for both Ca^2+^ and cAMP analysis, the equations were fit to the data after carbachol or bPAC stimulation, respectively.

## Results

### A Genetically-Encoded Reversible Biosensor to Detect Cell Stress

To assay cellular stress responses, we created a genetically encoded live-cell biosensor to detect an ER-mediated stress response through the IRE1α-XBP1 arm of the UPR. Versions of this type of biosensor have been previously described (Iwawaki et al., 2004; Roy et al., 2017), however, we sought to modify the sensor to adapt it for use in both imaging and plate reader-based assays. Upon detection of misfolded proteins within the lumen of the ER, IRE1α is activated and carries out an unconventional cytoplasmic splicing of the XBP1 transcript. This splicing leads to a frame shift in the XBP1 open reading frame, creating a functional transcription factor which in turn activates a host of stress response genes (Grootjans et al., 2016). Similar to previous versions of this biosensor, we co-opted this splicing event to shift into frame a bright green fluorescent protein, mNeonGreen (Figure 1A). Also in line with previous XBP1-based biosensors we either removed or maintained key sequence features of the XBP1 protein and fused this sequence to the mNeonGreen portion of the biosensor (Yanagitani et al., 2009; Peschek et al., 2015). These sequence features were either added or removed to create a biosensor that displayed similar activation and termination kinetics to the endogenous UPR. We first removed the DNA binding and basic domains of XBP1 as was done in previous versions of this biosensor (Iwawaki et al., 2004). Removing these domains from XBP1 is important as overexpression of full-length XBP1 inhibits the endogenous UPR response (Lee et al., 2003; Iwawaki et al., 2004). We also removed the leucine zipper domain of XBP1 to decrease the stability of the XBP1-mNeonGreen fusion. The leucine zipper domain is important for maintaining stability and expression of spliced XBP1 during the stress response (Uemura et al., 2013). Unspliced XBP1 protein enhances the degradation of the spliced protein. Binding of UBC9 to the leucine zipper domain inhibits this enhanced degradation (Uemura et al., 2013). By removing this domain from the portion of XBP1 that is fused to mNeonGreen, we created a biosensor that was likely sensitive endogenous UPR regulation. We term this modified XBP1-mNeonGreen fusion ΔXBP1-mNeonGreen. We also added a constitutively expressed red fluorescent protein upstream of the XBP1 intron to identify cells expressing the biosensor and as an indicator for protein expression levels. A self-cleaving 2A peptide was placed between the red and green fluorescent proteins to uncouple changes in protein expression from changes in ER-mediated cell stress. Finally, we targeted both fluorescent signals to the nucleus for easy image analysis and signal comparison. To test the ability of the biosensor to report on ER-mediated cell stress through the UPR, we compared the endogenous XBP1 splicing status to the biosensor splicing status during treatment with thapsigargin, a SERCA pump inhibitor that activates the IRE1α-XBP1 pathway. The splicing of the biosensor and the endogenous XBP1 transcript display similar profiles during a 24-h treatment with thapsigargin (Figure 1B). The highest level of spliced endogenous XBP1 transcript is observed between 2 and 6 h after thapsigargin treatment but diminishes within 24 h (van Schadewijk et al., 2012; Figure 1B). Both the splicing of the biosensor transcript and the stress-related green fluorescence peak between 2 and 6 h of treatment, diminishing after 22–24 h (Figures 1B–D), mimicking the endogenous stress response. Importantly, the cell stress response to thapsigargin is similar whether the stress levels are monitored using the fold change in green fluorescence on a plate reader (Figure 1C, top) or a high content imager using the percent of stressed cells within the well (Figure 1C, middle). Additionally, high content image analysis demonstrates that changes in cell stress occur an order of magnitude prior to changes to cell growth (Figure 1C, middle and bottom) when cells are stressed with thapsigargin. These data indicate that the cell stress biosensor may be more sensitive to detect changes in cellular stress response than assays monitoring cell growth or death.

An important feature of many neurodegenerative diseases is sustained cellular stress often caused by genetic mutations or changes in protein expression (Bosco et al., 2011; Ferrari et al., 2011). We next tested the ability of the cell stress biosensor to detect stress mediated through genetic mutations. We chose to assess a WT and mutant variant of rhodopsin. Rhodopsin is trafficked through the ER to the cell membrane in retinal cells (Deretic and Papermaster, 1991). Mutations in rhodopsin can lead to misfolding of the protein within the ER and are known to cause the progressive blinding disease, RP (Sung et al., 1991). By co-expressing the cell stress biosensor with either WT or a mutant form of rhodopsin known to cause RP, rhodopsin P23H, the effects of the WT and mutant rhodopsin were monitored over 20 h. Rhodopsin P23H not only displayed an increased cell stress response (Figure 1E, ΔF/F_green_ stress), it also resulted in decreased protein expression (Figure 1E, ΔF/F_red_ expression). This general decrease in protein expression is a hallmark of the UPR. These data demonstrate the ability of the cell stress biosensor to detect cell stress induced by genetic mutations. Lastly, as the cell stress biosensor may be a useful tool for assaying compounds that alleviate the cell stress response, we assessed the ability of the IRE1α inhibitor, 4μ8C to reduce the stress caused by the P23H mutation. After 20 h of expression, increasing doses of 4μ8C were added to cells expressing the P23H mutation and the subsequent changes in stress-related green fluorescence and expression-related red fluorescence were monitored independently (Figure 1F). As expected addition of 4μ8C reduced the stress response within 5 h after treatment (Figure 1F, ΔF/F_green_ stress), while no change in general protein expression was observed (Figure 1F, ΔF/F_red_ expression). Importantly, 1 μM of 4μ8C, which has an IC50 for IRE1α of 6.8 μM, was able to reduce stress levels of the P23H mutant back to those of WT within 20 h of treatment (Figure 1F, WT DMSO). These results demonstrate the ability of the cell stress biosensor to detect real-time changes in cell stress responses mediated by genetic components.

### Ca^2+^ Signaling Is Affected by ER Stress

Cellular stress responses, including those brought on by ER stress, can lead to a number of changes in cellular physiology. Second messenger signaling is especially sensitive to changes in cell state, thus we sought to explore how ER-mediated cell stress, brought on by the overexpression of WT and mutant P23H rhodopsin, affected intracellular Ca^2+^ signaling. We chose to explore Ca^2+^ signaling as the ER is the main store of Ca^2+^ within the cell, and changes to cytosolic Ca^2+^ has been associated with the P23H mutation (Shinde et al., 2016). We first created a modified baculovirus, BacMam, to express the cell stress biosensor along with the WT or mutant forms of rhodopsin. We then transduced HEK293T cells with increasing amounts of WT or mutant rhodopsin BacMam along with BacMam carrying the cell stress biosensor. Analysis of the stress signal, the expression signal, and the green/red ratiometric signal (Figure 2A) demonstrated a dose-dependent increase in cell stress mediated by WT and mutant P23H rhodopsin when compared to control HEK293T cells that were not transduced with either rhodopsin construct. The stress response from P23H rhodopsin became distinguishable from the WT stress response at 5 μl transduced virus, which corresponds to a similar level of rhodopsin mRNA expression observed in the retina (Table 1, Uhlén et al., 2015).

To determine if the stress response instigated by the P23H mutation affected Ca^2+^ signaling we co-transduced 5 μl of either WT or P23H rhodopsin BacMam along with the R-GECO Ca^2+^ biosensor (Wu et al., 2013) and the human cholinergic receptor muscarinic 1 (hM1) GPCR. Cells expressing either WT or P23H rhodopsin were then stimulated with carbachol to activate a Gq signaling response. Cells were imaged every 2 s before and during carbachol treatment. As shown in Figure 2B, the average Ca^2+^ response was quite different between the cells expressing WT and P23H rhodopsin. WT cells displayed an average ~1.75-fold change in cytoplasmic Ca^2+^ levels whereas P23H cells displayed only an average ~0.8-fold change. Inspection of individual Ca^2+^ traces from single cells imaged in Figure 2B suggested there may be distinct subpopulations of cells with variable responses to Gq activation. To explore this possibility, single-cell traces for the WT and P23H data were analyzed for Ca^2+^ response. First, each Ca^2+^ trace was fit to the following equation ΔF/F = αe^−θt^ to identify the Ca^2+^ amplitude (α) and cytoplasmic Ca^2+^ clearance rate (θ; Figure 2C). Cells expressing WT rhodopsin show two distinct populations of α values, with larger α values, indicating a larger Ca^2+^ signaling amplitude, dominating the distribution. Cells expressing P23H rhodopsin also display two distinct populations of α values. However, both populations are shifted to lower α values, indicating a blunted a Ca^2+^ signaling amplitude. Also, while a greater portion of cells display larger α values in the P23H expressing cells, the distribution is much more even between the two states than in WT cells (Figure 2C). Analysis of the distribution of values for θ from WT cells shows a single narrow distribution, while P23H expressing cells show a broader distribution of *θ* values. P23H cells also display a shift towards higher *θ* values, indicating a more rapid clearance of Ca^2+^ from the cytoplasm after hM1 stimulation (Figure 2C). The distinct distributions of α and θ from WT and P23H expressing cells support the notion that there may be subpopulations of cells with distinct Ca^2+^ signaling dynamics within the larger population of WT and P23H expressing cells. To explore this possibility, all single-cell traces from both the WT and P23H expressing cells were pooled and analyzed for the presence of unique Ca^2+^ signaling patterns. To identify the number of distinct Ca^2+^ signaling clusters present in the data, we tested the goodness of fit for 2–7 clusters of Ca^2+^ signaling profiles. Using the PBM index (Pakhira et al., 2004) to score cluster number, we identified four Ca^2+^ response profiles (Figure 2D). Two profiles, dominant in the WT cells, displayed increased Ca^2+^ responses upon hM1 activation (Figure 2E; profiles 3 and 4), resembling a typical Gq response (Tewson et al., 2013). The other two profiles, dominant in the P23H cells, displayed a blunted Ca^2+^ response (Figure 2E, profiles 1 and 2). However, the presence of profiles 1 and 2 in cells expressing WT rhodopsin suggests that even overexpression of WT rhodopsin elicits altered Ca^2+^ signaling along with increased ER stress (Figures 2A,C,E).

Analysis of ER-mediated cell stress and individual Ca^2+^ profiles suggests variability in Ca^2+^ signaling response may be related to varying levels of cellular stress induced by the rhodopsin P23H mutation. To explore this possibility, we created a modified version of the cell stress biosensor where the constitutively expressed red fluorescent protein was replaced with R-GECO (Figure 3A). We then co-expressed this version of the biosensor with rhodopsin P23H and hM1 and again activated Gq signaling through carbachol treatment. As seen in Figure 3B, cells with higher levels of cell stress had blunted Ca^2+^ responses, while those with lower levels of cell stress displayed a more typical Gq Ca^2+^ response. Comparing the fold change in Ca^2+^ levels upon Gq activation with stress levels revealed a negative correlation between cell stress and Ca^2+^ signaling. The higher the levels of cell stress, the more blunted the Gq Ca^2+^ response (Figure 3C). Together these data demonstrate that Ca^2+^ signaling dynamics are affected by ER-mediated cell stress. Moreover, the degree to which Ca^2+^ signaling is altered by cellular stress is dependent upon the severity of the cellular stress response.

### α-Synuclein Overexpression Induces Cell Stress and Alters PDE Activity

ER stress is also associated with protein folding diseases of proteins that are not directly trafficked through the ER (Scheper and Hoozemans, 2015). To test the ability of the cell stress biosensor to detect indirect ER stress, we created BacMam constructs of WT α-synuclein (α-syn) and four α-syn mutants associated with PD (Maiti et al., 2017). HEK293T and SH-SY5Y cells were co-transduced with BacMam containing the cell stress biosensor and either 1 μl or 10 μl of BacMam containing either WT or one of the mutant α-syn genes. SH-SY5Y cells were chosen along with HEK293T cells as they are a neuroblastoma cell line that has become an increasingly useful neuronal model to study not only PD but other neurodegenerative diseases as well (Nonaka et al., 2009; Xicoy et al., 2017; Vasquez et al., 2018). After 24 h of expression both the green fluorescence levels, to monitor stress induction, and the red fluorescence levels, to monitor protein expression, were analyzed using a plate reader. In both HEK293T and SH-SY5Y cells all of the α-syn mutants displayed increased ER-mediated cellular stress, compared to the WT at both 1 μl and 10 μl of virus (Figure 4A, green/red ratio). Notably, in both cell lines the α-syn mutants induced a significant decrease in overall protein expression at 1 μl of virus, which is equal to ~7× overexpression (Figure 4A red, Table 1). Normalizing the green stress fluorescent signal to this overall drop in expression results in significant detection of cell stress in each of the α-syn mutants. Interestingly, in HEK293T cells at 10 μl of the virus, the WT α-syn induces increased stress levels compared to the mutants, but in SH-SY5Y cells each α-syn mutant induced greater stress levels than the WT. This result suggests cell type may be an important factor to consider when assessing the effects of α-syn mutations on cellular responses. Together, these data demonstrate the ability of the cell stress biosensor to detect ER-mediated cell stress induced by genetic mutations affecting proteins in the cytoplasm as well as the ER.

Similarly to the rhodopsin mutant, we postulated that expression of α-syn variants might alter cellular signaling events along with inducing a stress response. Recent studies have identified phosphodiesterase (PDE) inhibitors as a way to preserve dopaminergic neurons (Morales-Garcia et al., 2011), promote neurogenesis (Morales-Garcia et al., 2015) and rescue Parkinsonian phenotypes (Bartolome et al., 2018). PDEs are key in the regulation of cyclic nucleotide second messenger levels within the cell as they degrade both cAMP and cGMP (Francis et al., 2011). We focused our analysis on cAMP as PDE inhibitor treatment increases cAMP levels within the cells which leads to increased CREB activation and expression of genes promoting neurogenesis (Morales-Garcia et al., 2011). How the regulation of basal levels of cAMP is altered in the disease state and how PDE activity affects cAMP dynamics in Parkinson’s models remains unclear. To address how changes in cell stress alter cAMP levels and PDE activity, we created a second live-cell assay to compare cell stress and cAMP signaling. We then assessed the effects of expressing different α-syn variants on cAMP levels and PDE activity in HEK293T and SH-SY5Y cells.

To remove the need for receptor activation to stimulate cAMP production, we developed a completely optical-based approach to monitor cAMP production and degradation. First, we used a blue light-activated adenylyl cyclase, bPAC (Stierl et al., 2011) to transiently raise cAMP levels within the cell, bypassing the endogenous receptor and adenylyl cyclase. The rapid off rate of bPAC allows direct monitoring of cAMP degradation by PDEs using R-cADDis, a red cAMP biosensor (Figure 4B). We first compared the PDE activity profiles between control cells and cells transduced with 1 μl of either WT or mutant α-syn, the lowest level of overexpression found to increase cellular stress. Initial analysis of cAMP degradation profiles revealed a marked difference in PDE activity in control cells and cells expressing any form of α-syn (Figure 4C). This trend was independent of the cell type analyzed as both HEK293T and SH-SY5Y cells expressing α-syn variants displayed similar increased PDE activity. We then determined the cAMP half-life (t_1/2_) in cells expressing different α-syn variants using the following logistic function, ΔF/F = α/(1+βe^−θt^). The distribution of cAMP t_1/2_ from individual cells expressing different α-syn variants was compared to the distribution of cAMP t_1/2_ determined from control cells. The cumulative distribution function for each α-syn variant and the control cells was then calculated and plotted to compare the cAMP t_1/2_ in both HEK293T and SH-SY5Y cells (Figure 4D). Consistent with the average cAMP degradation profiles, the α-syn variants displayed a shift towards increased PDE activity compared to control cells.

Taking advantage of the live-cell nature of this PDE activity assays, we inspected individual cAMP degradation profiles from cells expressing different α-syn variants. Interestingly, we identified multiple profiles of PDE activity within the cells. To identify the number of unique profiles and quantify their abundance in the different α-syn variants, we again used the PBM index score. In both HEK293T and SH-SY5Y cells, three unique cAMP degradation profiles were observed, rapid, intermediate, and delayed (Figures 5A,B). Overexpression of either WT or mutant forms of α-syn resulted in drastic changes in the cAMP degradation profile distribution in both cell types. In HEK293T cells, overexpression of any form of α-syn displayed a shift in cAMP degradation away from the delayed profile towards the rapid and intermediate profiles (Figures 5C,D). Similar shifts towards more rapid cAMP degradation were observed in SH-SY5Y cells as well (Figures 5E,F). These data are consistent with the increased cAMP degradation rates observed in the average profiles and cumulative distribution functions. Interestingly, in SH-SY5Y cells, the A53T and E46K mutations displayed an even greater shift towards the rapid profile when compared to WT α-syn (Figures 5E,F). The fact that overexpression of WT α-syn creates a shift towards increased PDE activity, with no observable change in ER-mediated cell stress, suggests that monitoring changes in cAMP degradation may be a more sensitive readout to detect defects in cellular function than ER-mediated cell stress. Or that changes to PDE activity may precede detectable changes in cell stress in overexpression models of WT α-syn. Notably, overexpression of WT α-syn has been shown to recapitulate Parkinson’s symptoms (Mochizuki et al., 2006; Chesselet, 2008). Together, these data suggest that in the presence of minimally overexpressed α-syn variants, cells undergo an ER-mediated cellular stress response and display increased PDE activity. These results are consistent with previous reports that PDE inhibitors can preserve dopaminergic neurons suggesting that cAMP levels play an important role in the progression of PD (Morales-Garcia et al., 2011, 2015).

### TDP-43 Overexpression Increases cAMP Degradation Rate

To determine if these changes in cell stress and cAMP degradation kinetics are unique to α-syn, we repeated the cell stress assay and optical interrogation of PDE activity using SH-SY5Y cells co-transduced with either WT or the M337V mutant of TDP-43. Overexpression of either WT or M337V versions of TDP-43 resulted in increased cell stress (Figure 6A) when compared to control cells at 1 μl of transduced virus, which corresponds to ~1.5× overexpression of TDP-43 in SH-SY5Y cells (Table 1). Transduction of 10 μl of virus, corresponding to ~5× overexpression, also resulted in increased cell stress when compared to control cells. Further, when compared to WT, the M337V mutation displayed a significant increase in cell stress at 10 μl of transduced virus (Figure 6A, 10 μl). Analysis of PDE activity again demonstrated a shift towards more rapid cAMP degradation in cells expressing WT or M337V TDP-43 compared to control cells (Figure 6B). Plotting the cumulative distribution function of cAMP t_1/2_ values from individual cells confirmed this shift towards more rapid cAMP degradation in cells expressing TDP-43 variants (Figure 6C). However, this shift was diminished compared to the shift observed in SH-SY5Y cells expressing α-syn variants (compare WT samples in Figures 4D, Figure 6C). These data suggest while TDP-43 overexpression increased PDE activity, it is to a lesser degree than α-syn overexpression. Lastly, analysis of cAMP degradation profiles by clustering revealed two distinct cAMP degradation patterns, a rapid and a delayed profile (Figure 6D). As control cells are analyzed along with cells expressing TDP-43 WT or TDP-43 M337V to determine cluster number, the intermediate profiles identified when comparing control cells to α-syn variants were segmented into either rapid or delayed profiles. Similarly to α-syn overexpression, both the WT and the M337V mutant of TDP-43 displayed a shift towards rapid cAMP degradation profiles at 1 μl of virus (Figures 6E,F), consistent with average cAMP degradation profiles and cumulative distribution functions. The M337V mutant also displayed a significant shift away from the delayed cAMP degradation profile towards the rapid profile when compared to WT TDP-43 (Figure 6F). These data are not only consistent with overexpression of TDP-43 increasing PDE activity, but with the notion that detectable changes in cell signaling are observed prior to changes in ER-mediated cell stress when comparing WT and M337V TDP-43 variants. These data, coupled with the similar results for α-syn, suggest that activation of cell stress and increased PDE activity are a common feature of overexpression of two of the major proteins associated with PD and ALS.

## Discussion

The cell stress biosensor described here builds upon previous attempts to create ER stress biosensors through the IRE1α-XBP1 pathway (Iwawaki et al., 2004; Roy et al., 2017). The generation of a reversible ER stress biosensor makes it possible to identify the onset as well as the alleviation of cell stress. This feature may prove useful for screening of compounds or genetic mutations that activate ER stress as well those that inhibit the response, using a single assay. The detection of ER stress brought on by a mutation in rhodopsin and the subsequent inhibition of this stress response by blocking IRE1α splicing activity through the use of 4μ8C is an intriguing example of such an intervention. Moreover, these assays were conducted in HEK293T cells but recapitulated the stress response observed in retinal cells. This type of assay, where both activation and inhibition of a cellular stress response can be detected in an immortalized cell model should prove useful. The genetic nature of these assays will also be useful in moving to *in vivo* models of neurodegeneration. *In vivo* models allow for a broader context for studying disease and having assays that can be deployed in both *in vitro* and *in vivo* models will help transition findings between the two. Thus, future work to optimize and deploy these assays in animal models will be an important next step.

Diseases such as RP, PD and ALS are difficult to study and treat because the assays used to study the effects of these diseases are often endpoint assays monitoring cell death. At this point, the ability to intervene and alter the course of the affected cell is lost. Furthermore, any changes in cell function, such as susceptibility to neurotransmitters and agonists, changes to second messenger regulation, or organelle function are best studied during the disease process. *In vitro* models for neurodegenerative diseases provide a useful platform to study these processes as many different mutations and cellular processes can be examined in a rapid manner. Studies of α-synuclein in SH-SY5Y cells highlight the usefulness of this *in vitro* system (Emadi et al., 2009; Xin et al., 2015; Xicoy et al., 2017; Vasquez et al., 2018). It is our hope that the biosensors and assays described here can be applied to these models to provide new insights into the changes in cellular function that accompany neurodegenerative-associated mutations. These assays may also provide a strategy to identify pertinent mutations, stress responses, and signaling events to be further assessed using *in vivo* models.

Many neurodegenerative diseases not only display cellular stress responses but changes in cellular signaling events as well (Xu et al., 2012; Shinde et al., 2016). The changes observed in cAMP and Ca^2+^ signaling induced by neurodegenerative mutations observed here also raises the question of how affected cells respond to stimuli. Changing the balance and timing of second messenger signaling in the cell may affect the ability of the cell to properly respond to, or transduce, extracellular signals such as neurotransmitters and neuromodulators. This alteration in cellular transduction status may also impact the druggability of cells. For example, a neuron expressing the A53T α-syn mutation displays an increased cell stress response and altered cAMP regulation. However, a neighboring cell, where the mutation has not caused build-up of misfolded protein yet, has intact cAMP regulation. If both cells are treated with agonists to the dopamine receptor there will likely be unique signal transduction responses in each cell. These differences are likely to affect how the cell responds or does not respond to the stimuli. These changes are important to consider in the development of drugs targeting these diseased cells. If a cell has lost its ability to transduce certain signals, those receptors, even though present at the cell surface, may no longer be a valid drug target. Indeed, our results suggest that targeting PDE activity may be a more effective method to restore the cellular transduction status. Thus, understanding how multiple stress and signaling pathways converge to affect cellular function in neurodegeneration will be an important step in the search for drugs to combat these diseases.

Finally, while ER-mediated cell stress is an important stress response involved in neurodegenerative disorders, many other cell stress pathways are involved. Proteasome stress, protein aggregation, mitochondrial stress, and oxidative stress have all been associated with neurodegenerative disease (Ciechanover and Kwon, 2017; Tan et al., 2019). Creating and combining genetically-encoded fluorescent biosensors for these pathways with biosensors for cellular signaling would further enhance our understanding of how neurodegenerative diseases disrupt cellular homeostasis and create new avenues for treatment of these diseases.

## Data Availability Statement

All raw images and CellProfiler analysis scripts are available on figshare at: https://doi.org/10.6084/m9.figshare.9209486.

## Author Contributions

KH and TH designed the experiments and wrote the manuscript. ER conducted RT-qPCR, RT-PCR, viral transductions and cell culture. SM produced BacMam virus. KH conducted cell culture, transfections and transductions, collected imaging data, plate reader data, and analyzed the data. JC conducted high content experiments and analysis.

## Conflict of Interest

KH and TH hold a provisional patent filing for cell stress biosensors coupled to cell signaling biosensors. KH, TH, ER, and SM were employed by Montana Molecular. JC was employed by BioTek Instruments Inc.

## Footnotes

^1^www.r-project.org
